# Stability, dissolution, and cytotoxicity of NaYF_4_-upconversion nanoparticles with different coatings

**DOI:** 10.1038/s41598-022-07630-5

**Published:** 2022-03-08

**Authors:** Verónica Bastos, Párástu Oskoei, Elina Andresen, Maysoon I. Saleh, Bastian Rühle, Ute Resch-Genger, Helena Oliveira

**Affiliations:** 1 Department of Biology and CESAM, University of Aveiro, 3810-193 Aveiro, Portugal; 2 BAM Federal Institute of Materials Research and Testing, Division Biophotonics, Richard-Willstätter-Str. 11, 12489 Berlin, Germany; 3 Institut für Chemie und Biochemie, Physikalische und Theoretische Chemie, Freie Universität Berlin, Takustraße 3, 14195 Berlin, Germany; 4 Department of Chemistry, Faculty of Science, The University of Jordan, Amman, 11942 Jordan

**Keywords:** Chemistry, Materials science, Nanoscience and technology

## Abstract

Upconversion nanoparticles (UCNPs) have attracted considerable attention owing to their unique photophysical properties. Their utilization in biomedical applications depends on the understanding of their transformations under physiological conditions and their potential toxicity. In this study, NaYF_4_:Yb,Er UCNPs, widely used for luminescence and photophysical studies, were modified with a set of four different coordinatively bound surface ligands, i.e., citrate, alendronate (AA), ethylendiamine tetra(methylene phosphonate) (EDTMP), and poly(maleic anhydride-alt-1-octadecene) (PMAO), as well as silica coatings with two different thicknesses. Subsequently, the aging-induced release of fluoride ions in water and cell culture media and their cytotoxic profile to human keratinocytes were assessed in parallel to the cytotoxic evaluation of the ligands, sodium fluoride and the lanthanide ions. The cytotoxicity studies of UCNPs with different surface modifications demonstrated the good biocompatibility of EDTMP-UCNPs and PMAO-UCNPs, which is in line with the low amount of fluoride ions released from these samples. An efficient prevention of UCNP dissolution and release of cytotoxic ions, as well as low cytotoxicity was also observed for UCNPs with a sufficiently thick silica shell. Overall, our results provide new insights into the understanding of the contribution of surface chemistry to the stability, dissolution behavior, and cytotoxicity of UCNPs. Altogether, the results obtained are highly important for future applications of UCNPs in the life sciences and bioimaging studies.

## Introduction

Spectrally converting, near-infrared (NIR)-excitable, lanthanide doped nanoparticles or upconversion nanoparticles (UCNPs) with their multi-color emission have emerged as promising candidates for advanced biomedical applications such as bioimaging and sensing, photodynamic therapy, triggered release of drugs by heat and light, neuromodulation, immunotherapy and optogenetics^1–6^. NaYF_4_ is one of the most widely used host lattices for UCNPs and is generally considered to yield the most efficient inorganic upconverting crystalline materials^7^ with commonly assumed excellent chemical stability^8–10^ and very low solubility^11^.

Despite the large number of advantages of UCNPs and possible biomedical applications, concerns about their biosafety and potential adverse effects have been raised^12,13^, as the dissolution of NaYF_4_ UCNPs in aqueous media can result in the release of fluoride and lanthanide ions which can induce cytotoxicity in biological systems^14–18^, particularly under high dilution conditions in aqueous media^11,14,15,19^. Surface modification with various ligands has been used not only to tune the dispersibility and colloidal stability of NaYF_4_ UCNPs, but also to reduce their dissolution in aqueous environments, as the release of fluoride and lanthanide ions may induce cytotoxicity in biological systems^14–18,20^. For example, surface coatings such as amphiphilic, layer-by-layer or polysulfonate coatings were shown to decelerate UCNP dissolution by preventing water molecules to access the particle surface^18,19,21,22^.

Recently, the role of five different surface coatings, namely poly(acrylic acid (PAA), polyallylamine (PAH), polyethylene glycol (PEG), citrate, and *N*,*N* bis(phosphonomethyl)glycine (PG)) was evaluated by Plohl et al.^18^. These authors also found that UCNP dissolution could be significantly decreased by a PMAO–BHMT coating. Also, more significant dissolution of the UCNPs was observed in PBS than in water. Himmelstoß et al.^23^ reported that negatively charged UCNPs surface-modified with citrate, PAA, and PG did not show a good resistance against dissolution, as transmission electron microscopy (TEM) images showed changes in UCNP morphology and size. They also observed higher dissolution rates in highly diluted systems commonly used in biological applications with mass concentrations of 100 µg/mL or lower. The dissolution of these particles was accompanied by a constant drop in the upconversion luminescence (UCL) intensity, that can also be used to monitor changes in particle surface chemistry and UCNP size as demonstrated in previous works^15,24,25^, and an increase in the hydrodynamic diameter due to UCNP aggregation. For PEG groups, the weaker binding of the ether groups to the UCNP surface induced a deformation of the NPs, accompanied by a decrease in luminescence by 30%. In contrast, functionalization with positively charged PAH led to a remarkable colloidal and chemical stability at higher mass concentrations (5000 µg/mL) with a slight change of morphology and a small change in UCL intensity for lower concentrations (1000 µg/mL)^23^.

In our previous work, we assessed the Y^3+^ and F^−^ release from 20 and 30 nm NaYF_4_:20%Yb^3+^, 2%Er^3+^ UCNPs surface-stabilized with citrate and polyacrylic acid (PAA) or coated with mesoporous or microporous silica shells of different thickness over time, at room temperature and at 37 °C in water, PBS and culture medium^26^. Moreover, the stability of NaYF_4_:20%Yb^3+^, 2% Er^3+^ UCNPs stabilized with the phosphonate coatings alendronate and EDTMP in PBS at room temperature and 37 °C was also investigated^25^. This revealed enhanced UCNP dissolution by a reduced particle size, an elevated temperature, and PBS and its deceleration with phosphonate ligands or a sufficiently thick silica shell.

In the present work, as an expansion, we address the effect of size and surface modification of typical NaYF_4_:Yb^3+^, Er^3+^ UCNPs on their cytotoxicity profile and the relation to UCNP stability and dissolution behavior. Therefore, the stability and cytotoxicity of hexagonal NaYF_4_:Yb,Er cores with a size of 20 nm were assessed for the following surface modifications: (i) uncoated (bare), coated with (ii) citrate ligands, (iii) ethylendiamine tetra(methylene phosphonate) (EDTMP), (iv) alendronate (AA), (v) poly(maleic anhydride-alt-1-octadecene) (PMAO), vi) thin silica shell (10 nm) and vii) thick silica shell (73 nm). To roughly examine the effect of size, NaYF_4_:Yb,Er cores with 30 nm were also synthesized and the oleate capping agents remaining on the UCNP surface from the synthesis were removed to yield uncoated (bare) nanoparticles, as well as particles stabilized with citrate after a ligand exchange. Special emphasis was put on possible cytotoxic effects of these differently surface functionalized UCNPs on human HaCaT keratinocyte cells. This cell line was selected as a sensitive model for assessing the cytotoxicity of different types of surface modified UCNPs, as previously described by Guller et al.^27^. To also take contributions from the surface ligands and released ions to overall cytotoxicity into account, similar studies were done with the surface ligands as well as with fluoride and lanthanide ions at concentrations estimated to be released on the human HaCaT keratinocyte cells during UCNP dissolution.

## Materials and methods

### Chemicals

Lanthanide chlorides with high purity (99.99%), sodium oleate (82%), oleic acid and octadecene (90%, both technical grade), hexadecyltrimethylammonium bromide (CTAB, 98%), citric acid (99.5%), diethylene glycol (DEG, 99%), N,N-dimethyl formamide (DMF, 99.5%), nitrosyl tetrafluoroborate (95%), polyacrylic acid (MW = 1800 Da), trisodium citrate dihydrate (95%), tetraethyl orthosilicate for synthesis (98%), ammonia solution (25% wt% in water), and *trans*-1,2-diaminocyclohexane-N,N,N′,N′-tetraacetic acid monohydrate were purchased from Sigma Aldrich (Germany). Ethylenediamine tetra(methylene phosphonic acid) (EDTMP) (98%) was obtained from ABCR GmbH and polyoxyethylene bis(amine) M.W. 1000 and alendronic acid (97%) were obtained from Alfa Aesar GmbH. Poly(maleic andydride-alt-octadecene) M_n_ 30,000–50,000 was obtained from Sigma Aldrich (Germany). All chemicals were used without further purification. All solvents employed for the optical measurements were purchased from Sigma Aldrich in spectroscopic grade. Water refers here to Milli-Q water.

Regarding chemicals and reagents for cell culture, dimethyl sulfoxide (DMSO; ≥ 99.7%) and CCK-8 reagent were purchased from Sigma-Aldrich (St. Louis, MO, USA). The HaCaT cell line, a nontumorigenic immortalized human keratinocyte cell line (Boukamp et al., 1988), was obtained from CLS Cell Lines Services (CLS Cell Lines Service Eppelheim, Germany). Dulbecco’s Modified Eagle’s Medium, fetal bovine serum (FBS), l-glutamine, fungizone (250 U mL^−1^) and trypsin–ethylendiaminetetraacetic acid (EDTA) (0.25% trypsin and 1 mM EDTA) were purchased from Gibco, Life Technologies (Grand Island, NY, USA). Penicillin–streptomycin (10,000 U mL^−1^) was purchased from Grisp (Porto, Portugal).

### Synthesis and surface modification of UCNPs

The synthesis of UCNPs composed of NaYF_4_ doped with 20% Yb^3+^ and 2% Er^3+^ was performed according to previously reported procedure with some modifications. The preparation of ligand-free (HCl route), alendronate, EDTMP coated (indirect route) and citrate coated UCNPs (procedure 2) were performed according to protocols previously described in^25^, and the syntheses of ligand-free UCNPs (BF_4_-coated), citrate coated (procedure 1) and silica shelled UCNPs were performed as previously described in^26^. The respective surface modification procedures have been detailed in previous publications from us^25,26^.

*Amphiphilic coatings*: 1 mL of UCNP dispersion in cyclohexane (c = 16 mg/mL) was precipitated with acetone, centrifuged at 3000 rpm for 5 min, and then redispersed in 1.5 mL of chloroform. 30 mg of PMAO was dissolved in 0.5 mL of chloroform and added to the UCNP dispersion. The dispersion was stirred for 1 h at rt.

For NaYF_4_:YbEr@PMAO: The UCNP-PMAO dispersion in chloroform was evaporated to dryness and 2 mL of 0.1 M NaOH were added. The resulting UCNP dispersion was stirred for 1 h at 60 °C to hydrolyze the anhydride groups. The NaYF_4_:YbEr@PMAO nanoparticles were collected by centrifugation (30 min at 10,000 rpm, 13,639 rcf) and washed three times with Milli-Q water. Finally, the NaYF_4_:YbEr@PMAO nanoparticles were redispersed in 2 mL of Milli-Q water (c = 8 mg/mL) and stored at 5 °C.

For NaYF_4_:YbEr@PMAOcross: The UCNP-PMAO dispersion in chloroform was evaporated to dryness and re-dispersed in 2 mL of THF. Five milligrams of PEG-diamine were added to the dispersion and the reaction solution was stirred over night at rt. The dispersion was evaporated to dryness and 2 mL of 0.1 M NaOH were added. The dispersion was stirred for 1 h at rt to hydrolyze the remaining anhydride groups. NaYF_4_:YbEr@PMAOcross UCNPs were collected by centrifugation (30 min at 10,000 rpm, 13,639 rcf) and washed three times with Milli-Q water. Finally, the NaYF_4_:YbEr@PMAOcross nanoparticles were redispersed in 2 mL of Milli-Q water (c = 8 mg/mL) and stored at 5 °C.

### Characterization

Transmission electron microscopy (TEM) images were obtained with a Talos F200S Microscope (Thermo Fisher Scientific) using an accelerating voltage of 200 kV. The samples were prepared by placing a droplet of UCNP dispersions (1 mg/mL in water) onto a 3 mm copper grid (lacey, 400 mesh) and letting it dry under air at rt. The average particle size was determined from 2 to 3 micrographs (58 kx magnification) of approximately 500 particles. The area of the particles was automatically measured using a fixed threshold based on the image intensity histograms and the size distribution descriptors (Feret_max_ and Feret_min_) were determined. The obtained diameters were plotted as histograms and fitted with a Gaussian curve. The mean (μ) and standard deviation (σ_x_) of this curve were taken as the representative particle size for the respective sample.

FT-IR measurements of the coated particles were acquired on a Nicolet Nexus FT-IR spectrometer (Thermo Electron Corporation) using an ATR accessory. The spectra were recorded in a wavenumber range of 4000–400 cm^−1^.

Thermal gravimetric analysis (TGA) of the coated particles was performed using a Hitachi STA 7200 set-up with Sample Changer AS3. TGA experiments were performed over a temperature range of 30–600 °C under nitrogen atmosphere (200 mL/min), switching to synthetic air at 600 °C (200 mL/min), and using a heating rate of 10 K/min.

### Steady state and time-dependent emission measurements

Spectrally resolved UCL measurements were carried out on an Edinburgh Instruments Model FLS980-xD2-stm spectrofluorometer equipped with an 8 W, 978 nm laser diode. The emission wavelength range was set to 500–700 nm and the slit width was 5 nm.

Luminescence decay measurements used as an indirect method to assess particle stability were carried out on an Edinburgh Instruments Model FLS980-xD2-stm spectrofluorometer equipped with an electrically pulsed, 8 W, 978 nm laser diode (long square pulses, pulse width of 150 μs). The decay kinetics were recorded at 540 nm, 655 nm (Er-UC), and 1000 nm (Yb-DC) with a red-sensitive photomultiplier tube (PMT; Model H10720-20) from Hamamatsu, using time-correlated single photon counting (TCSPC).

### Particle aging and released fluoride measurement

Particle aging experiments were performed by dispersing UC-bare-20 (BF_4_) and UC-SiO_2_-thick at 50 µg/mL in 10 mL of Mili-Q water or DMEM, followed by vortex mixing for 72 h at room temperature. After that, the solutions were centrifuged for 1 h at 13,080 rcf and the top 9 mL of supernatant were collected. For the released fluoride quantification, UCNPs were dispersed at a concentration of 50 µg/mL followed by further incubation at 37 °C for 24 h and 48 h. At selected time points, the solutions were centrifuged, the supernatant was collected with a pipette, and the amount of fluoride ions in the supernatant was determined with an ion-selective electrode from Metrohm with an integrated temperature sensor using the Tiamo 2.4 Light Software. The measurements were performed in freshly prepared TISAB IV concentrate containing 10 mM trans-1,2-diaminocyclohexane-N,N,N',N'-tetracetic acid (CDTA), 1 M sodium chloride and 1 M acetic acid. Sodium hydroxide was used to adjust pH at around 5.5.

### In vitro cell culture and cell viability assay

HaCaT cells were grown in high glucose DMEM medium supplemented with 10% fetal bovine serum (FBS), 2 mM L-glutamine, 100 U/mL penicillin, 100 μg/mL streptomycin and 2.5 μg/mL fungizone at 37 °C in a humidified atmosphere with 5% CO_2_. Cells were daily observed for confluence and morphology using an inverted phase-contrast Eclipse TS100 microscope (Nikon, Tokyo, Japan).

Cell viability was determined by the WST-8 assay using the CCK-8 reagent. HaCaT cells were seeded in 96-well plates at 60,000, 40,000, and 20,000 cells/well for 24 h, 48 h and 72 h of exposure, respectively. The cells were exposed to the NaYF_4_ UCNPs listed in Table 1 at different concentrations (0; 12.5; 25; 50; 100 and 200 μg/mL). As control, the cells were also exposed to the surface ligands or lanthanide salts and sodium fluoride for 24 and 48 h. The concentration range of the ions and surface ligands used in the cytotoxicity assays was calculated as the upper limit of the amounts that can maximally be released at particle concentrations of 6.25 µg/mL (i.e., half of the lowest particle concentration we tested), at 12.5 µg/mL (i.e., the lowest particle concentration we tested), 100 µg/mL (i.e., half of the highest particle concentration we tested), 200 µg/mL (i.e., the highest particle concentration we tested), and 400 µg/mL (i.e., twice the highest particle concentration we tested). The calculations and estimations are explained in more detail in the “Results and discussion” section. In the aging experiments, the cells were exposed to collected culture medium where UCNP-bare-20 (BF_4_) and UC-SiO_2_-thick at 5 and 50 µg/mL were incubated for 72 h, as described above. In case of aging of UCNPs in H_2_O, the solutions were freeze dried and then resuspended in culture medium before cell exposure. Cells were exposed to the different aging solutions and further incubated for 72 h. The exposure of the cells was followed by incubation at 37 °C in 5% CO_2_ humidified atmosphere for the previously indicated periods. At the end of the incubation, the medium was removed and 100 μL of culture medium were added together with 10 μL of CCK-8 reagent to each well and incubated for 2 h at 37 °C in 5% CO_2_. The absorbance at 450 nm was measured with a microplate reader (Synergy HT Multi-Mode, BioTek, Vinooski, VT).

**Table 1:** NaYF_4_:Yb,Er surface modifications studied, including surface coating, size and the abbreviations used in the text.

UCNP Sample	Surface Coating	Diameter (nm)	Abbreviations
Bare NaYF_4_:YbEr	BF_4_	20	UC-bare-20 (BF_4_)
Bare NaYF_4_:YbEr	BF_4_	30	UC-bare-30 (BF_4_)
Bare NaYF_4_:YbEr	HCl	20	UC-bare-20 (HCl)
NaYF_4_:YbEr@citrate	Citrate (procedure 1)	20	UC-citrate-20 (P1)
NaYF_4_:YbEr@citrate	Citrate (procedure 1)	30	UC-citrate-30 (P1)
NaYF_4_:YbEr@citrate	Citrate (procedure 2)	20	UC-citrate-20 (P2)
NaYF_4_:YbEr@silica thick	silica	20 (shell thickness: 73 nm)	UC-SiO_2_-thick
NaYF_4_:YbEr@silica thin	silica	20 (shell thickness: 10 nm)	UC-SiO_2_-thin
NaYF_4_:YbEr@Alendronate	Alendronate	20	UC-AA-20
NaYF_4_:YbEr@EDTMP	EDTMP	20	UC-EDTMP-20
NaYF_4_:YbEr@PMAO	PMAO	20	UC-PMAO-20
NaYF_4_:YbEr@PMAO cross	PMAO cross	20	UC-PMAO cross-20

### Statistical analysis

The results are reported as mean ± standard deviation (SD) of 3 technical replicates in each of the 3 independent experiments for 24 h and 48 h. For all the assays, the statistical significance between control and exposed cells was performed by one-way ANOVA, followed by Dunnet and Dunn’s method (as parametric and non-parametric test, respectively), using Sigma Plot 14 software (Systat Software Inc.). The differences were considered statistically significant from *p* < 0.05.

## Results and discussion

### UCNP surface modification and spectroscopic characterization

UCNP surface modifications used in this study and their respective abbreviations are summarized in Table 1. Except for the PMAO-based coating, the synthesis and characterizations of these UCNPs have been previously reported^25,26^.

**Figure 1:**
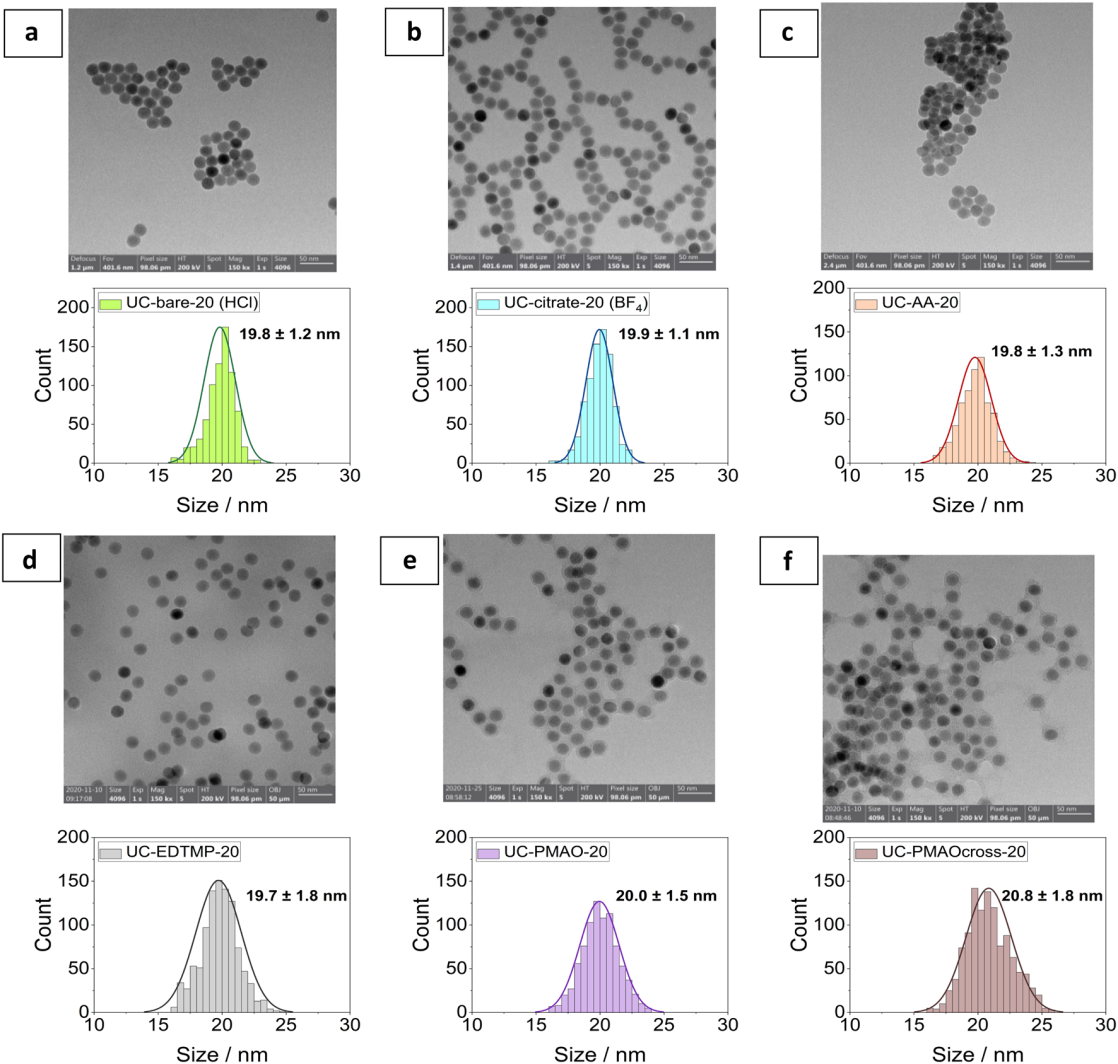
Transmission electron microscopy (TEM) images of UCNP with different surface coatings. (**a**) UC-bare-20 (HCl); (**b**) UC-citrate-20 (BF_4_); (**c**) UC-AA-20; (**d**) UC-EDTMP-20; (**e**) UC-PMAO-20; (**f**) UC-PMAOcross-20.

**Figure 2:**
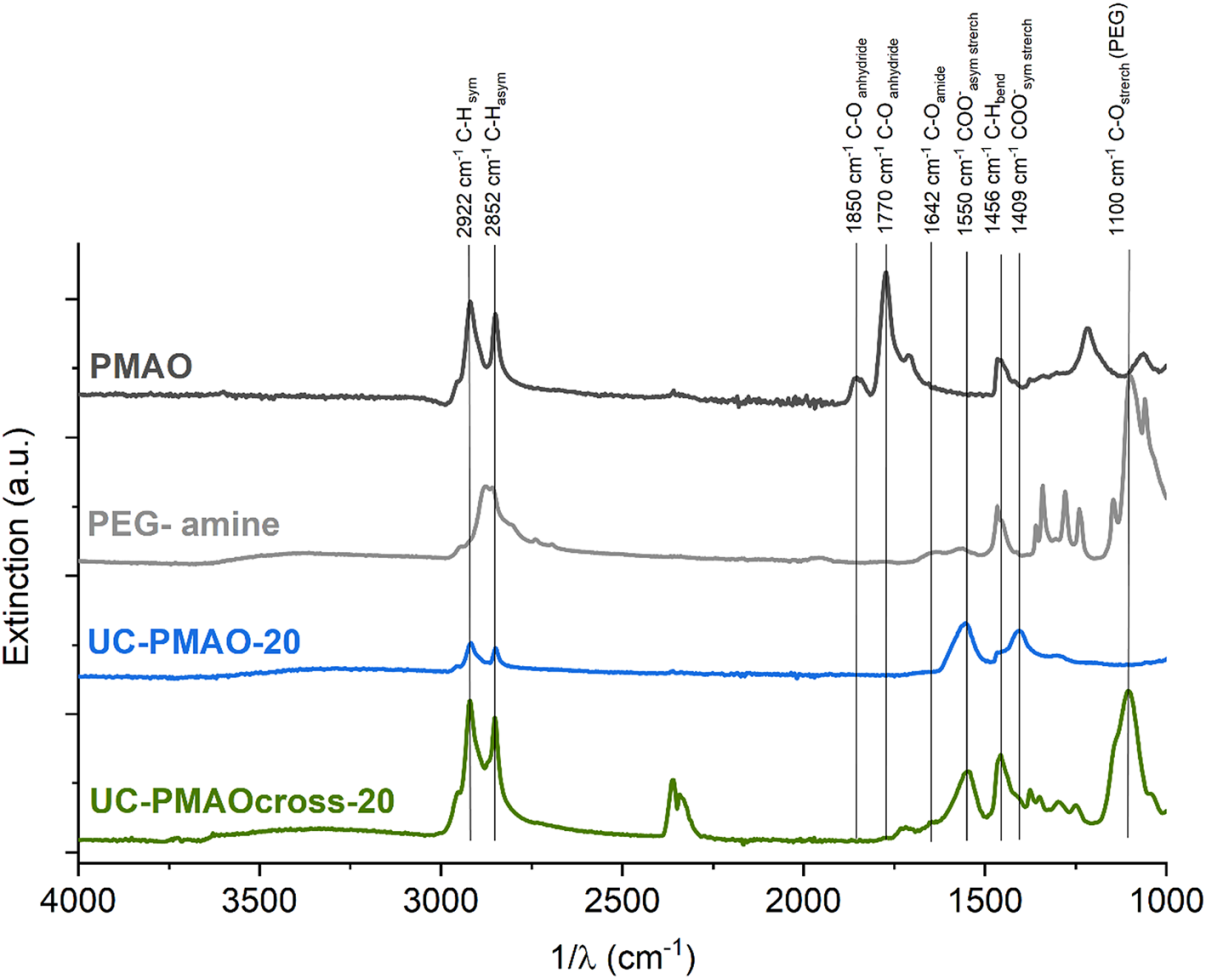
ATR-FTIR spectra of UCNPs with amphiphilic coatings and the corresponding ligands.

To confirm the uniformity of the particles regarding particle size, morphology and shape after ligand exchange and surface functionalization, TEM images of selected samples are presented in Fig. 1 and in reference^26^. FTIR measurements were used to evaluate the ligand exchange for the phosphonate coatings and were previously reported except for the amphiphilic coatings^25^. ATR-FTIR spectra obtained for UC-PMAO-20 and UC-PMAO cross-20 nanoparticles, illustrated in Fig. 2, verified the formation of amphiphilic coatings on the UCNP surface. For a better comparison, the spectra of the free PMAO and the crosslinker PEG-diamine are also illustrated. For unhydrolyzed PMAO, the characteristic absorption peaks at 1770 cm^−1^ and 1865 cm^−1^ represent the succinic anhydride C=O symmetric and asymmetric stretching vibration, respectively. After hydrolysis, the peaks at 1770 cm^−1^ and 1850 cm^−1^ from the anhydride disappear, and more carboxylate groups are formed, as evidenced by the peaks corresponding to the carboxylate C=O stretching at 1550 cm^−1^ and 1409 cm^−1^. The amidation reaction between the carboxylic acids (from PMAO anhydride rings) and the amine group (from PEG-NH_2_) was evidenced from the peak at around 1642 cm^−1^ corresponding to the C=O vibration of the amide bond. The NH deformation vibration expected in the region of 1515–1570 cm^−1^ and the NH stretch vibration expected at around 3100–3500 cm^−1^ are difficult to identify due to the overlap with the COO^−^ and the OH vibrations, respectively. For the UC-PMAO cross-20 nanoparticles, the characteristic peaks at around 1200–1400 cm^−1^ (C–H bending vibration) and 1100 cm^−1^ (C–O stretching vibration) are observed, providing an additional hint for the successful cross-linking process.

**Figure 3:**
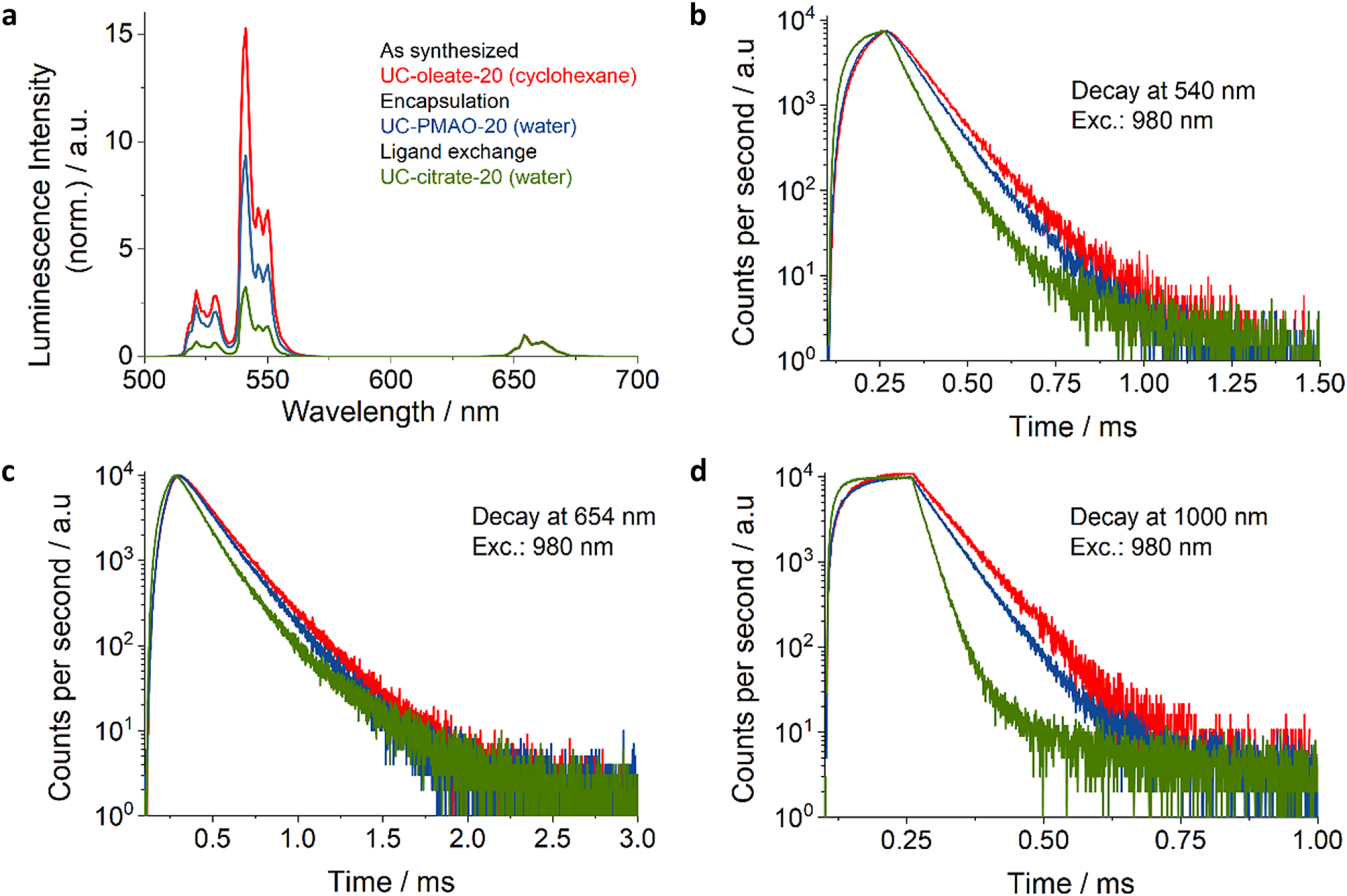
Luminescence properties of the differently coated hydrophilic UCNPs modified via encapsulation (blue) and ligand exchange (green) compared to the as-synthesized hydrophobic UCNPs. (**a**) Photoluminescence spectra measured at an excitation power density (*P*) of 22 W/cm^2^ using a 980 nm laser diode; the spectra were normalized at 654 nm. (**b**) Lifetime of the Er^3+^ upconversion luminescence (UCL) excited at 980 nm (*P* of 67 W/cm^2^; pulse width 40 µs) and recorded at 540 nm; (**c**) Lifetime of the Er^3+^ UCL excited at 980 nm (*P* of 128 W/cm^2^; pulse width 40 µs) recorded at 655 nm; and d) Lifetime of the downshifted luminescence (DSL) of Yb^3+^ excited at 980 nm (*P* of 128 W/cm^2^; pulse width 40 µs) and recorded at 1000 nm.

Ligand exchange and phase transfer to an aqueous solvent can affect the luminescence properties of UCNP^24^ as the efficiency and relative spectral distribution of UCL is governed by the competition between the energy transfer upconversion (ETU) rates from the Yb^3+^ sensitizers to the Er^3+^ activators and the depopulation rates of the intermediate states of the activator ions involved in the UC process^24^. Efficient coupling of the fundamental vibrational modes of water molecules (located between about 3300 to 3700 cm^−1^) at or near the UCNP surface to the Yb^3+^ and Er^3+^ energy levels favors the multiphoton deactivation of the Yb^3+^ sensitizer and the non-radiative depopulation of the ^4^S_3/2_/^2^H_11/2_ and ^4^I_11/2_ energy levels of Er^3+^^28^. Water-induced luminescence quenching is exemplarily shown for 21.5 nm sized UCNPs in Fig. 3. This also provides information on the accessibility of the UCNP surface to water molecules which can initiate particle decomposition resulting in the release of potentially toxic fluoride and lanthanide ions. Figure 3 displays the luminescence spectra (normalized to the red Er^3+^ luminescence band at 654 nm) and lifetimes of the excited energy levels of Yb^3+^ and Er^3+^ of as-synthesized oleate capped UCNPs in cyclohexane (UC-oleate-20) and UCNPs with hydrophilic surface modifications highlighting the corresponding changes of the intensity ratios of the green and red upconversion emission bands (see also Table 2). As shown in Fig. 3, the intensity of the green emission at 540 nm drops by a factor of ∼ 3 for the UCNPs obtained via ligand exchange and by a factor of ~ 2.5 and ~ 1.9 for UCNP coated with amphiphilic molecules and amphiphilic polymers, respectively. The effect of water quenching is also reflected by the decay kinetics and reduced lifetimes of the downshifted luminescence (DSL) of Yb^3+^ at 1000 nm and the green and red upconversion luminescence of Er^3+^ (see also Table 2). These results indicate that the amphiphilic coatings, obtained via an encapsulation strategy involving the intercalation with the hydrophobic oleate ligands shield the UCNP surface more effectively from water molecules as compared to surface modifications accompanied by an exchange of the hydrophobic surface ligands.

**Table 2:** Spectroscopically determined parameters for differently coated UCNPs.

Surface coating	Green-to-red intensity ratio	Luminescence lifetime at		
		1000 nm	540 nm	654 nm
Oleate	15.3	85.4 µs	105.7 µs	164.0 µs
Ligand-free or small ligand e.g. Citrate	3.0	38.6 µs	80.2 µs	119.0 µs
PMAO coatings	8.5	77.2 µs	102.8 µs	159.7 µs

^a^Intensity-weighted lifetime:${{\varvec{\tau}}}_{{\varvec{i}}{\varvec{n}}{\varvec{t}}}=\frac{{{\varvec{A}}}_{1}{{\varvec{\tau}}}_{1}^{2}+{{\varvec{A}}}_{2}{{\varvec{\tau}}}_{2}^{2}}{{{\varvec{A}}}_{1}{{\varvec{\tau}}}_{1}+{{\varvec{A}}}_{2}{{\varvec{\tau}}}_{2}}$

### Fluoride release during particle aging

In our previous works, we studied the dissolution of UC-bare-20 (BF_4_), UC-bare-30 (BF_4_), UC-citrate-30 (P1), UC-SiO_2_-thick and UC-SiO_2_-thin over time (6 h to 72 h), at different temperatures (RT and 37 °C), and in different solvents (water, PBS and culture medium)^26^, and examined the fluoride release from UC-bare-20 (HCl), UC-AA-20 and UC-EDTMP-20 in PBS at RT and 37 °C^25^. In this study, we focused on the fluoride release from different NaYF_4_ UCNPs at 37 °C in water and cell culture medium after 24 h and 48 h incubation to relate the fluoride release from the different NaYF_4_ UCNPs with the putative cytotoxicity in vitro. The fluoride release from the different bare and surface modified NaYF_4_ UCNPs in water and DMEM culture medium at 37 °C after 24 h and 48 h of incubation is summarized in Table 3. Fluoride release after 48 h in water was higher for UC-citrate-20 (P1) and lower for UC-SiO_2_-thick. As previously shown, a silica shell can protect UCNPs from dissolution over time, with the protective effect increasing with increasing shell thickness. Also, EDTMP capping protected UCNP from fluoride release. UCNP incubation in cell culture medium, led to a substantial decrease in fluoride release for UC-bare-20 (BF_4_), UC-citrate-20 (P1), and UC-SiO_2_-thin compared to fluoride release in water. This was ascribed to the formation of a protective layer of adsorbed molecules from the DMEM culture medium on the UCNP surface^26^.

**Table 3:** Measurement of released fluoride [µM] from the different UCNPs in water and DMEM culture medium at 37 °C after 24 h and 48 h incubation. n.d.: not determined.

UCNPs	F-Release 37 °C Water [µM] after 24 h	F-Release 37 °C Water [µM] after 48 h	F-Release 37 °C DMEM [µM] after 24 h	F-Release 37 °C DMEM [µM] after 48 h
UC-bare-20 (BF_4_)	239.47	297.37	5.79	5.79
UC-citrate-20 (P1)	263.68	330.53	14.21	16.84
UC-SiO_2_-thin	100.58	164.26	4.74	8.42
UC-SiO_2_-thick	3.16	4.42	8.42	11.58
UC-bare-20 (HCl)	124.39	156.32	n.d.	n.d.
UC-AA-20	94.39	123.46	n.d.	n.d.
UC-EDTMP-20	26.49	25.30	n.d.	n.d.

### Cytotoxicity studies

Mandatory for all biomedical applications of NPs like UCNPs is their biocompatibility, requiring their toxicological evaluation^13^. Generally, the cytotoxicity of NPs depends on particle parameters like morphology, size, hydrophobicity, and surface charge. As the ions constituting UCNPs and the surface ligands could be released in physiological media and biological environments upon UCNP disintegration, it is important to also address the contribution of dissolved UCNP constituents to the toxicity of UCNPs. In addition, the surface coating must be assessed regarding its protective role on nanoparticle dissolution and its potential toxicity. To our knowledge, despite some research on the dissolution of UCNPs^17,18,25,26^, the impact of these combined effects on UCNP toxicity has not yet been systematically investigated.

**Figure 4:**
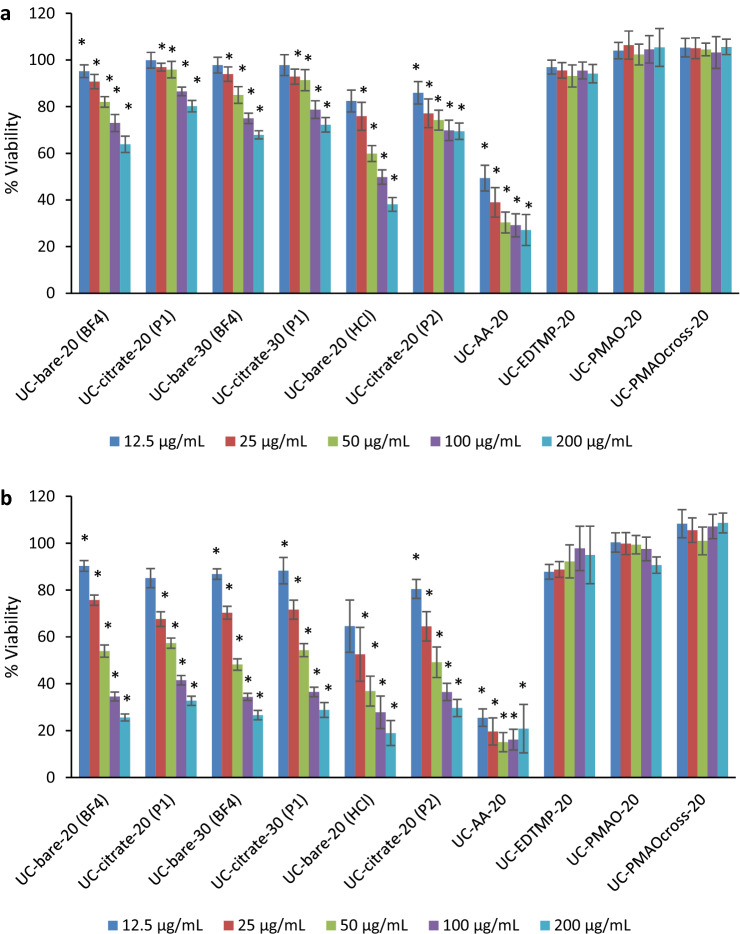
Viability of HaCaT cells after exposure to UCNPs with different surface coatings for (**a**) 24 h and (**b**) 48 h: UC-bare-20 (BF4); UC-citrate-20 (P1); UC-bare-30 (BF4); UC-citrate-30 (P1); UC-bare-20 (HCl); UC-citrate-20 (P2); UC-AA-20; UC-EDTMP-20; UC-PMAO-20; UC-PMAOcross-20. The results are reported as mean ± standard deviation (SD) of 4 technical replicates in each of the 3 independent experiments for 24 h and 48 h. * indicates significant statistically differences compared to the control (p < 0.05).

**Figure 5:**
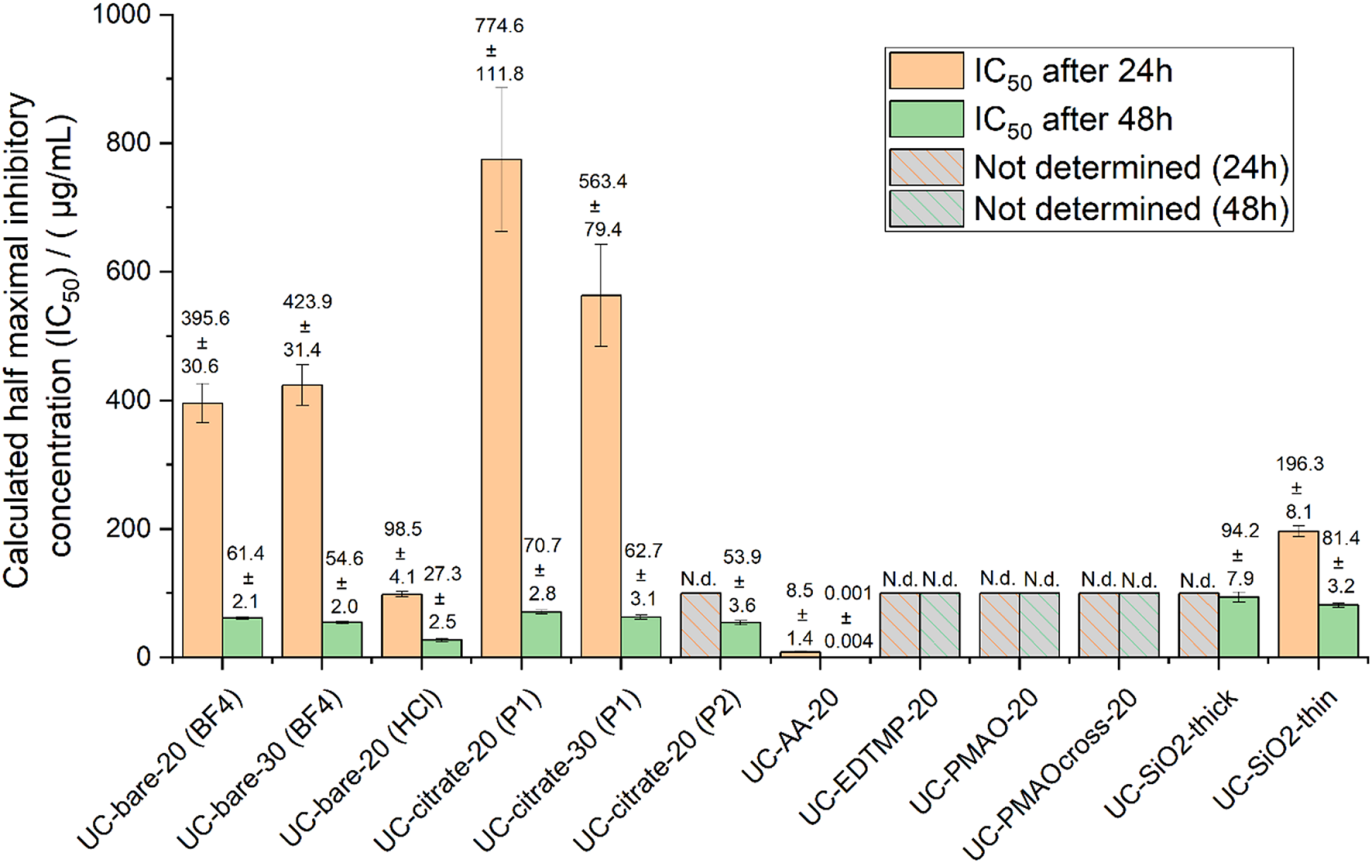
Calculated half maximal inhibitory concentration (IC50) at 24 h and 48 h exposure with UCNPs of different surface modification. N.d.: not determined.

The cytotoxicity of the different NaYF_4_ UCNPs, as well as their surface ligands alone and their constituting ions in the form of the respective salts was evaluated in human HaCaT keratinocyte cells utilizing the WST-8 assay. For bare and citrate coated UCNPs, the effect of size and the protocol used for ligand stripping and citrate coating on the toxicity was also assessed. According to the data provided in Fig. 4, citrate functionalized (“procedure 1” and “procedure 2”) UCNPs exhibited lower cytotoxicity than the bare UCNPs obtained via the “BF_4_^−^ procedure” and “HCl procedure”. As the surface charge plays a significant role in the cellular uptake and cytotoxicity of nanoparticles^29^, this is attributed to the positive surface charge of the bare UCNPs revealing a positive zeta potential of about + 40 mV while the citrate coated UCNPs have a negative zeta potential of about − 40 mV. For example, a higher cytotoxicity was reported for positively charged silica nanoparticles^30^, silica-titania hollow nanoparticles^31^, and gold nanoparticles^32^ as compared to their negatively charged analogs. Regardless of the presence or absence of surface functionalization and size, the cytotoxicity was dose dependent and increased with time, being more pronounced after 48 h (Fig. 4b). Figure 5 and Table S1 show the calculated half maximal inhibitory concentration (IC_50_) for the studied UCNPs. While exhibiting similar trends for ligand free and citrate coated UCNPs, our results indicate that a citrate coating reduces the cytotoxicity. Bare UCNPs were slightly (“BF_4_^−^ procedure”) or markedly (“HCl procedure”) more cytotoxic than citrate coated UCNPs, as shown by the lower IC_50_ values. However, no clear trend can be established for the effect of UCNP size on the cytotoxic profile. The IC_50_ at 24 h exposure for the 20 nm UCNPs was 395.6 µg/mL for UC-bare-20 (“BF_4_^−^ procedure”), 98.5 µg/mL for UC-bare-20 (“HCl procedure”), and 774.6 µg/mL for UC-citrate-20 (“procedure 1”). For the 30 nm UCNPs the estimated IC50 at 24 h were 423.9 µg/mL and 563.4 for UC-bare-30 (“BF_4_^−^ procedure”) and UC-citrate-30 (“procedure 1”), respectively. After 48 h of exposure, these nanoparticles induced a similar toxicity, i.e., the observed differences in the cytotoxic potential are clearly diminished, as confirmed by very similar IC50 values.

Regarding the cytotoxicity of the organophosphonate capped UCNPs, the alendronate coated (UC-AA-20) were the most cytotoxic to HaCaT cells, presenting very low IC_50_ values both at 24 h (8.49 µg/mL) and 48 h (0.001 µg/mL), contrarily to EDTMP coated UCNP, which did not affect the cell’s viability at tested concentrations. Absence of toxicity was also found for cells exposed to UCNPs functionalized with either PMAO or crosslinked PMAO at the tested dose range.

**Figure 6:**
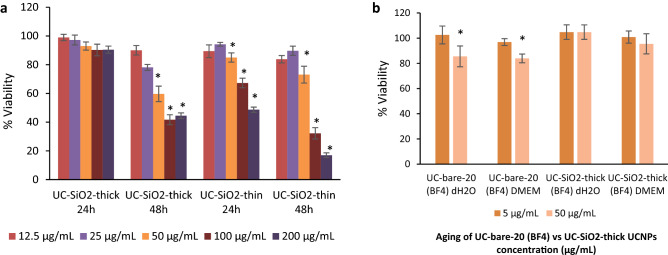
Viability of HaCaT cells exposed to (**a**) UC-SiO_2_-thick and UC-SiO_2_-thin for 24 h and 48 h; and (**b**) UC-bare-20 (BF_4_) and UC-SiO_2_-thick after 72 h of aging in di-H_2_O and DMEM. The results are reported as mean ± standard deviation (SD) of 4 technical replicates in each of the 3 independent experiments for 24 h and 48 h. The differences were considered statistically significant from *p* < 0.05: * indicates significant differences compared to the control.

To investigate a possible relation between the degree of cytotoxicity, silica shell thickness, and particle stability, UCNPs from the same particle batch bearing thin and thick silica shells were examined. As shown in Fig. 6a, after 24 h of exposure, UC-SiO_2_-thick can be considered biocompatible to HaCaT keratinocytes up to the highest concentration of 200 µg/mL tested in this study (cell viability = 90.45 ± 2.47). In contrast, UC-SiO_2_-thin induced a significant viability decrease from 50 µg/mL even for 24 h of exposure (Fig. 6a). For incubation times increased to 48 h, both types of silica shelled UNCPs decreased the cell viability. Still, UC-SiO_2_-thin were more cytotoxic than UC-SiO_2_-thick, especially for higher concentrations. Our results indicate that thick silica shells may confer some protection to ions release compared to a thin shell or the uncoated UCNPs. Therefore, to compare the toxicity of the different UCNPs to the HaCaT keratinocyte cells to that originating from constituting ions released during nanoparticle aging, UC-SiO_2_-thick and UC-bare-20 were incubated for 72 h in di-H_2_O and DMEM cell culture medium. Ion release after 72 h by UC-bare-20 at 50 µg/mL in Mili-Q water and cell culture medium induced a significant decrease in HaCaT cell viability, while the aging solutions of UC-SiO_2_-thick did not affect cell viability (Fig. 6b). This is in accordance with the lower toxicity of UCNPs with thicker silica shells as compared to those with thinner silica shells previously reported by Kembuan et al.^12^ for macrophages (RAW 264.7 cells).

**Figure 7:**
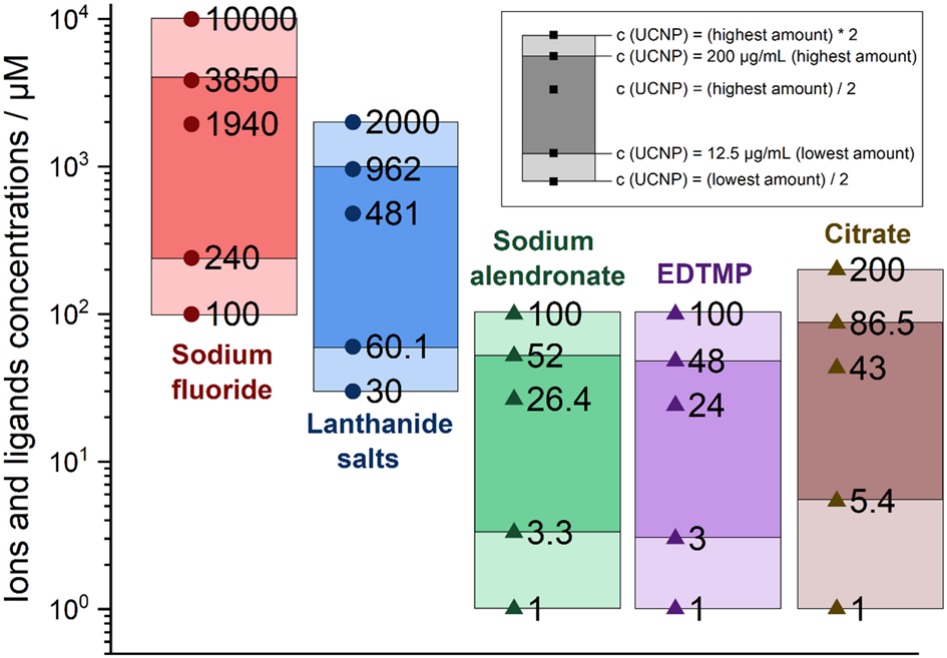
Concentration range of ions and ligands (µM) used for viability tests based on calculations of their total amount present in the lowest (12.5 µg/mL) and highest (200 µg/mL) concentration of UCNPs for the studied particles, assuming their complete release.

**Figure 8:**
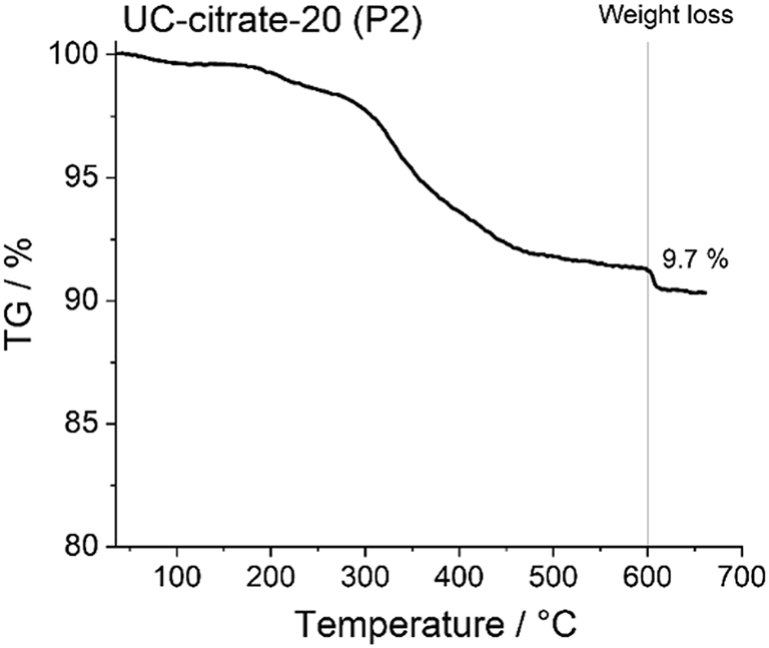
Thermogravimetric analysis (TGA) curves of 21.5 nm-sized NaYF4:Yb,Er particles with citrate coating (procedure 2).

**Figure 9:**
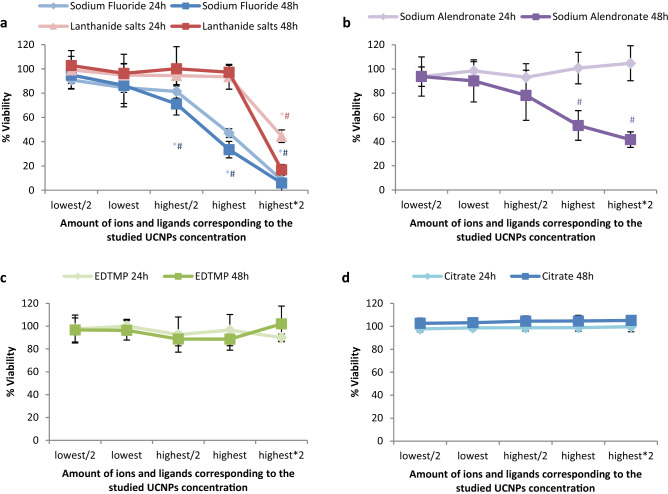
Viability of HaCaT cells after exposure to the UCNP constituting ions and ligands at concentrations matching the concentration range of released upon exposure of the UCNPs; the lowest amount (amount present in the 12.5 µg/mL of UCNPs), half of the lowest amount, half of the highest amount, highest amount (amount present in the 200 µg/mL of UCNPs), and twice of the highest amount. a) Sodium fluoride and lanthanide salts; b) Sodium alendronate; c) EDTMP; d) Citrate. The results are reported as mean ± standard deviation (SD) of 4 replicates from each of the 3 independent experiments for 24 h and 48 h. The differences were considered statistically significant from *p* < 0.05: * indicates significant differences compared to the control after 24 h, # after 48 h.

Considering that the cytotoxic effects of UCNPs may be due to the release of ions from UCNP cores or ligands from the UCNP surface, the influence of the amount of ions and ligands present on the UCNP surfaces on cell viability was also examined for different UCNP concentrations (Fig. 7 and Table S2). As previously reported based on TGA measurements^25^ for the phosphonate coatings, the approximate amount of alendronate and EDTMP ligand for the 21.5 nm sized UCNPs is 0.37 µmol/mg and 0.28 µmol/mg, respectively. For the citrate coated UCNPs (UC-citrate-20 P2), TGA measurements showed a mass loss of 9.7%, which corresponds to 0.51 µmol of citrate per mg of particles (Fig. 8). We estimated upper limits for the maximally possible amount of ion release (assuming complete particle dissolution) from the stoichiometric UCNP formula NaY_0.78_Yb_0.20_Er_0.02_F_4_ and a bulk density of 4.21 g/cm^3^, and the maximum amount of ligands introduced by the particles from TGA measurements (assuming complete release of all ligands from the particle surface and attributing all weight loss observed in TGA to organic surface ligands). We did these calculations and estimations for the following particle concentrations: 6.25 µg/mL (i.e., half of the lowest particle concentration we tested), 12.5 µg/mL (i.e., the lowest particle concentration we tested), 100 µg/mL (i.e., half of the highest particle concentration we tested), 200 µg/mL (i.e., the highest particle concentration we tested), and 400 µg/mL (i.e., twice the highest particle concentration we tested), and then assessed the cytotoxicity of the free ligands and ions (see Fig. 7, Table S2 and Fig. 9). Figure 4 showed that UC-bare-20 (HCl) induced significant viability decrease from 25 µg/mL, whereas the concentration corresponding to the maximum of lanthanide ions release did not induce cytotoxicity (Fig. 9a). This suggests that lanthanide ions, despite being heavy metals, are not the main driver for cytotoxic effects of UCNPs on HaCaT cells upon dissolution. However, sodium fluoride induced a significant viability decrease from the concentration corresponding to half of the maximum used in UCNPs which is equivalent to the concentration released at 100 µg/mL of UCNPs under the assumption of complete dissolution. These results are in line with the results reported by Chang et al.^16^ and Salomão et al.^20^, which indicate cytotoxicity induction by fluoride ions released from UCNPs. To get around this issue, surface modifications are being reported to decelerate or even inhibit the dissolution of UCNPs, resulting in a reduction of the negative impacts on cell viability. A positive effect of particle coating regarding cell viability was also found by Rafique et al.^33^ when comparing the toxicity of bare and PAA coated UCNPs, who found that the latter are less cytotoxic to HeK293, HeLa, A549, SCC7 cell lines.

Although alendronate coated UCNPs (UC-AA-20) showed a significant cytotoxicity already at low doses (Fig. 4), the alendronate (AA) ligand itself only induced a significant viability decrease for the highest concentration used for UC-AA-20 and for exposure times of 48 h (Fig. 9b). Moreover, fluoride release during dissolution of UC-AA-20 in water after 24 h (Table 3) was comparable to the fluoride release observed for UC-bare-20 (HCl) (94.39 µM and 124.39 µM, respectively). These results indicate that the cytotoxicity observed for UC-AA-20 can partially be explained by the toxicity of alendronate, mainly after 48 h, combined with the low protection against particle dissolution conferred by this ligand. Regarding the citrate coated UCNPs, their toxicity profile can most likely be ascribed to released fluoride ions due to incomplete protection against dissolution from citrate coated UCNPs, as citrate itself is a very biocompatible surface ligand (Fig. 9d) and is not cytotoxic for concentrations as high as 200 µM (172 µM representing twice the maximum concentration present on the UCNPs at the highest concentration tested). These results are also in agreement with previous studies^34,35^.

UCNPs coated with EDTMP revealed a good biocompatibility up to 48 h of exposure and high nanoparticle concentration (200 µg/mL) (Fig. 4). When considering the toxicity of the EDTMP ligand, results showed very good biocompatibility at concentrations that correspond to twice the concentration present in the highest tested dose (Fig. 9c). Also, the EDTMP coating proved to be very effective in preventing fluoride release. Regarding the biocompatibility of the PMAO coating, our results showed that this ligand itself did not affect cell viability (Fig. 4), which agrees with the good biocompatibility of PMAO coated UCNPs. Similar response profiles were observed by Plohl et al.^18^ who found that UCNPs@PMAO-BHMT were biocompatible to EAhy926 endothelial cells up to 200 µg/mL. Also, Guller et al.^27^ reported ~ 80% viability of dermal fibroblasts and keratinocytes (HaCaT), after 24 h exposure to PMAO coated UCNPs at 62.5 and 125 µg/mL, supporting the biosafety of PMAO surface-modified UCNPs for theranostic applications on human skin.

## Conclusion and outlook

The future utilization of UCNPs in biomedical and life science applications requires an in-depth understanding of their transformation under physiological conditions and their potential cytotoxicity. In this work, we have shown that both bare and citrate coated UCNPs induced a time and concentration dependent decrease in cell viability, with the citrate coated particles being less cytotoxic than the bare UCNPs. The toxicity of citrate coated UCNPs can most likely be attributed to the fluoride ions released by partial nanoparticle dissolution, as citrate itself is a highly biocompatible surface ligand. Alendronate coated UCNPs were the most cytotoxic to HaCaT cells, and their cytotoxicity can partially be explained by the toxicity of alendronate itself, combined with the low protection conferred by this ligand against particle dissolution. EDTMP coated UCNPs showed no cytotoxic effects to HaCaT cells, which agrees very well with the improved protection provided by the EDTMP ligand against fluoride release and its good biocompatibility. Absence of toxicity was also found for cells exposed to UCNPs functionalized with either PMAO or crosslinked PMAO.

Regarding the role of a silica shell on UCNP’s dissolution and toxicity, we have shown that the silica shell seems to confer stability to UCNPs and protect them from dissolution over time. This protective effect increases with an increase in shell thickness, as reflected by lower fluoride release. UCNPs with thicker silica shell were less cytotoxic than those with thinner shells, especially at higher UCNP concentrations. Aged solutions of bare UCNPs induced toxicity to HaCaT cells, contrarily to thicker silica shelled particles, confirming the association between dissolution behavior and toxicity.

In summary, our work provides valuable insights into the biocompatibility of UCNPs and the required surface modifications for further biological applications. In the future, we plan to do similar studies with differently sized UCNPs, particularly smaller ones, to assess whether the coatings identified as optimum in this study are also suitable to provide the desired biocompatibility for UCNP with sizes below 20 nm. Moreover, we will examine whether luminescence studies, providing information on the accessibility of water molecules to the surface of UCNPs, can be correlated with UCNP stability and particle disintegration in aqueous environments like bioanalytically relevant buffers.

## Supplementary Information

Supplementary Information.
