# Supplementary material for: Stability, dissolution, and cytotoxicity of NaYF4-upconversion nanoparticles with different coatings
Source: Sci Rep. 2022 Mar 8;12:3770. doi: 10.1038/s41598-022-07630-5 (PMC8904531; doi:10.1038/s41598-022-07630-5)
Supplement: Supplementary file 1 — Supplementary Information. [file 41598_2022_7630_MOESM1_ESM.docx]

Supplementary Information

**Stability, Dissolution, and Cytotoxicity of NaYF_4_-Upconversion Nanoparticles with Different Coatings**

Verónica Bastos^1^, Párástu Oskoei^1^, Elina Andresen^2^, Maysoon I. Saleh^2,3,4^, Bastian Rühle^2^, Ute Resch-Genger^2*^, Helena Oliveira^1*^

1 - Department of Biology and CESAM, University of Aveiro, 3810-193 Aveiro, Portugal

2 - BAM Federal Institute of Materials Research and Testing, Division Biophotonics, Richard-Willstätter-Str. 11, D-12489 Berlin, Germany

3- Institut für Chemie und Biochemie, Physikalische und Theoretische Chemie, Freie Universität Berlin, Takustraße 3, D-14195 Berlin,Germany.

4- Department of Chemistry, Faculty of Science, The University of Jordan, Amman, 11942, Jordan.

* Corresponding author e-mails: ute.resch@bam.de; holiveira@ua.pt

*

**Table S1:** Calculated half maximal inhibitory concentration (IC_50_) at 24h and 48h exposure with UCNPs of different surface modification. N.d.: not determined

| UCNPs | IC_50_ ± SD after 24h (µg/mL) | IC_50_ ± SD after 48h (µg/mL) |
| --- | --- | --- |
| UC-bare-20 (BF_4_) | 395.6±30.56 | 61.4±2.06 |
| UC-bare-30 (BF_4_) | 423.9±31.42 | 54.6± 2.03 |
| UC-bare-20 (HCl) | 98.5±4.14 | 27.3± 2.53 |
| UC-citrate-20 (P1) | 774.6±111.79 | 70.7±2.79 |
| UC-citrate-30 (P1) | 563.4±79.36 | 62.7±3.07 |
| UC-citrate-20 (P2) | N.d. | 53.9± 3.56 |
| UC-AA-20 | 8.5±1.44 | 0.001± 0.004 |
| UC-EDTMP-20 | N.d. | N.d. |
| UC-PMAO-20 | N.d. | N.d. |
| UC-PMAOcross-20 | N.d. | N.d. |
| UC-SiO_2_-thick | N.d. | 94.2 ± 7.90 |
| UC-SiO_2_-thin | 196.3 ± 8.08 | 81.4 ± 3.23 |

**Table S2**: Concentration range of ions and ligands (µM) used on viability tests based on calculations of their total amount present in the lowest (12.5 µg/mL) and highest (200 µg/mL) concentration of UCNPs for the studied particles, assuming their complete release. The concentration range used was: the lowest amount, half of the lowest amount, highest amount, half of the highest amount and twice of the highest amount.

|  |  | **Sodium Fluoride** | **Lanthanide salts** | **Sodium alendronate** | **EDTMP** | **Citrate** |
| --- | --- | --- | --- | --- | --- | --- |
| **UCNP concentrations** | **12.5 µg/mL (lowest amount)** | 240 µM | 60.1 µM | 3.3 µM | 3 µM | 5.4 µM |
|  | **200 µg/mL (highest amount)** | 3850 µM | 962 µM | 52 µM | 48 µM | 86.5 µM |
| **Ions and ligands concentrations** | **Half of the lowest amount** | 100 µM | 30 µM | 1 µM | 1 µM | 1 µM |
|  | **Lowest amount** | 240 µM | 60.1 µM | 3.3 µM | 3 µM | 5.4 µM |
|  | **Half of the highest amount** | 1,950 µM | 481 µM | 26.4 µM | 24 µM | 43 µM |
|  | **Highest amount** | 3,850 µM | 962 µM | 52 µM | 48 µM | 86.5 µM |
|  | **Twice of the highest amount** | 10,000 µM | 2000 µM | 100 µM | 100 µM | 100 µM |
